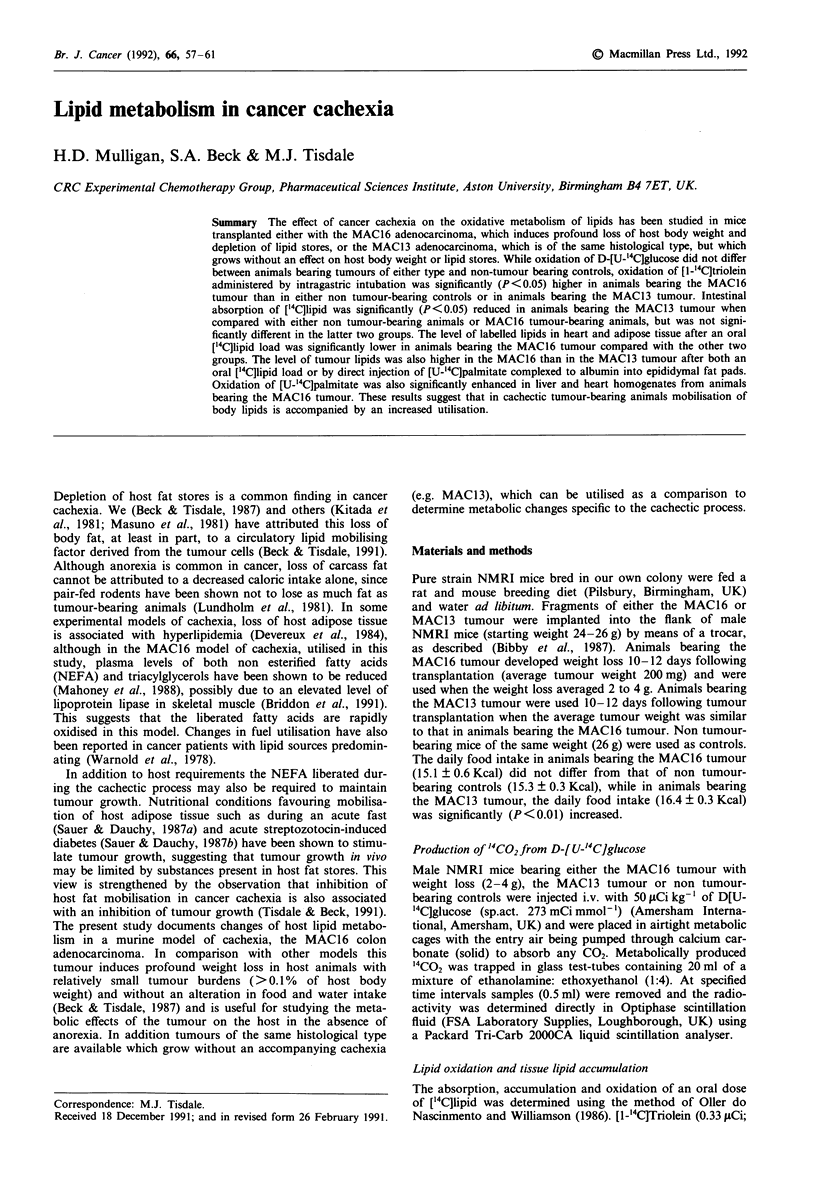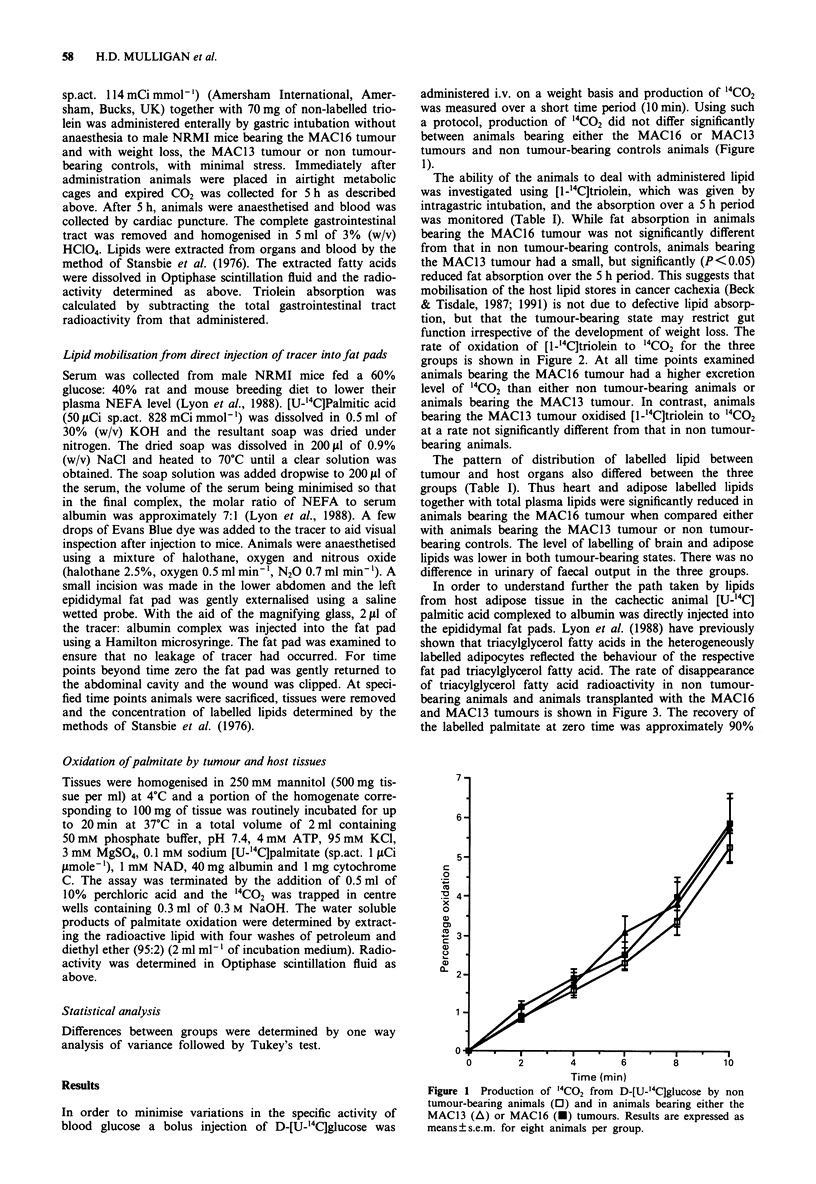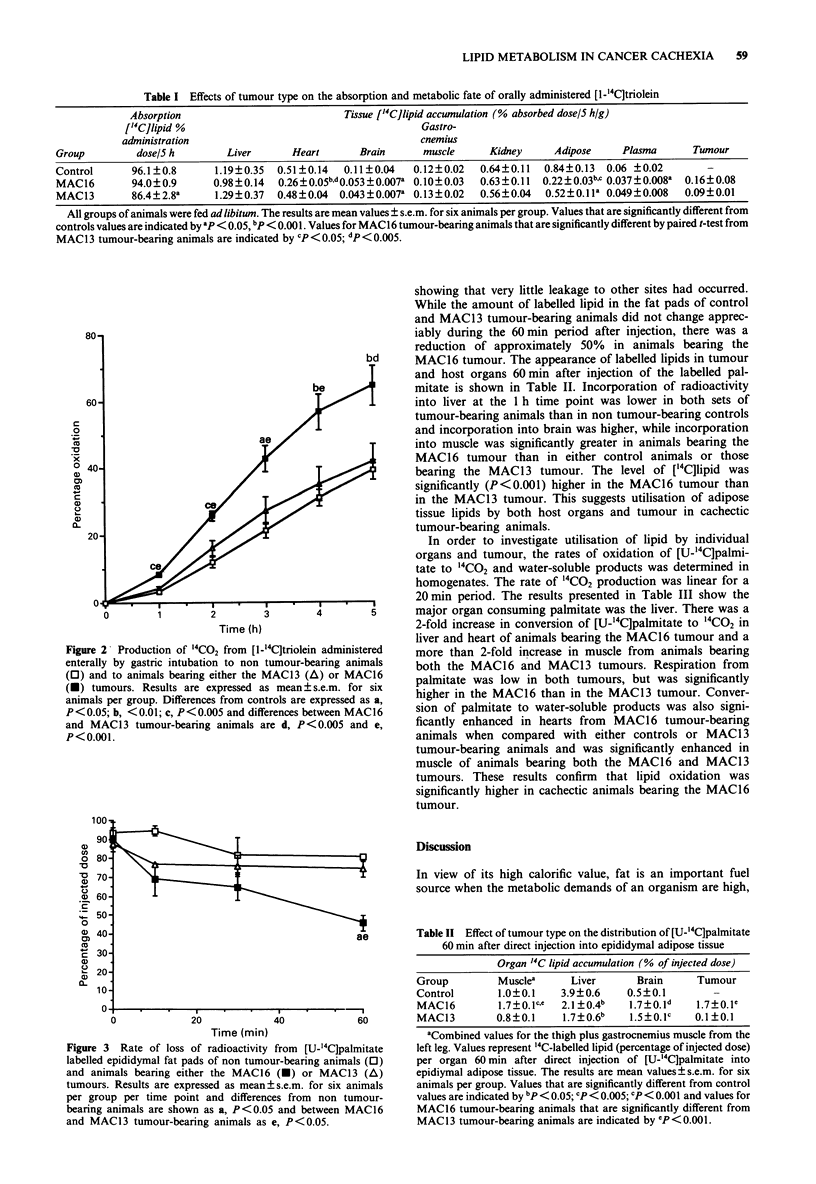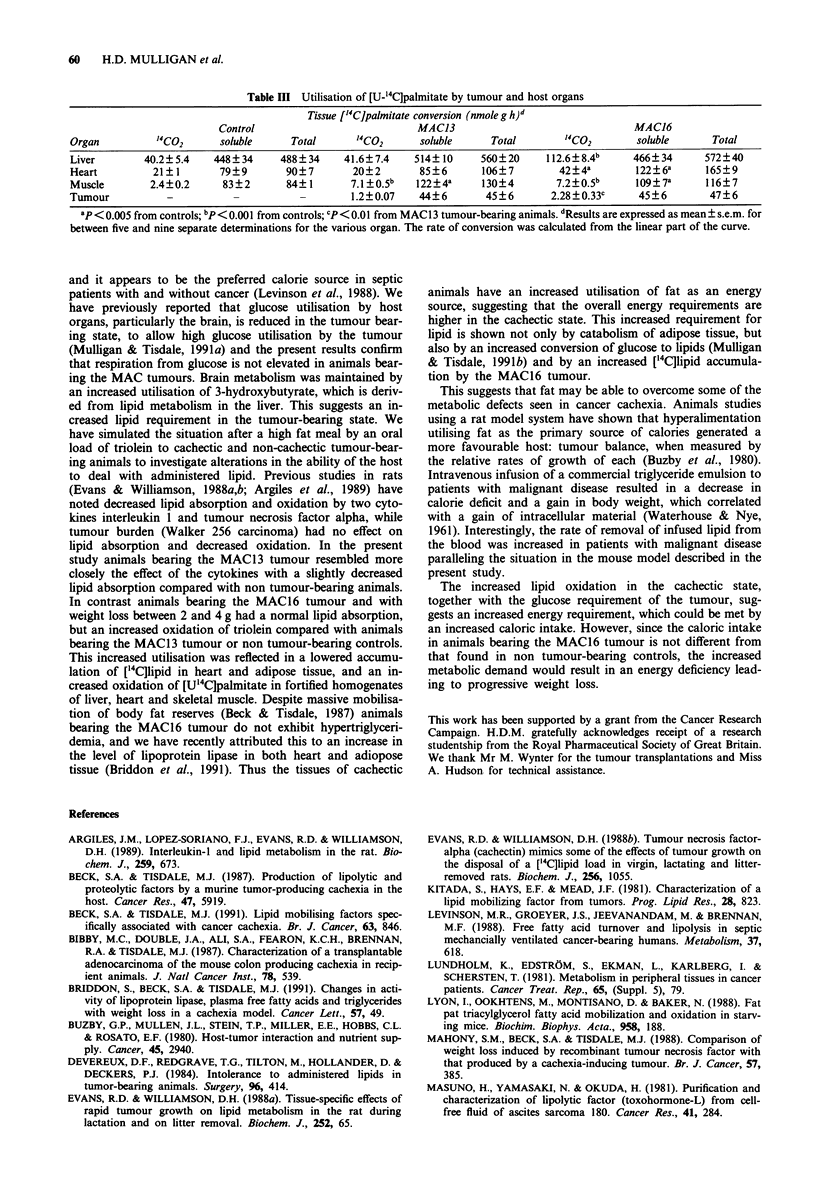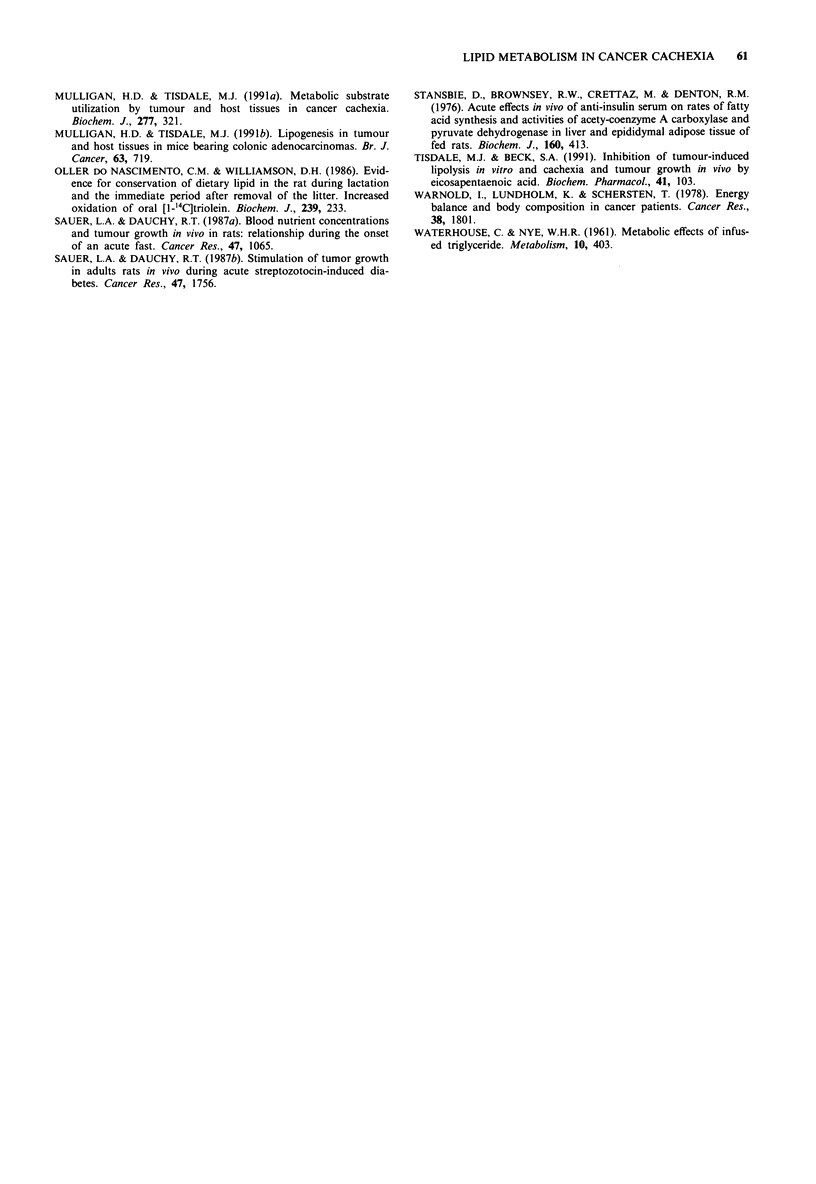# Lipid metabolism in cancer cachexia.

**DOI:** 10.1038/bjc.1992.216

**Published:** 1992-07

**Authors:** H. D. Mulligan, S. A. Beck, M. J. Tisdale

**Affiliations:** CRC Experimental Chemotherapy Group, Pharmaceutical Sciences Institute, Aston University, Birmingham, UK.

## Abstract

The effect of cancer cachexia on the oxidative metabolism of lipids has been studied in mice transplanted either with the MAC16 adenocarcinoma, which induces profound loss of body weight and depletion of lipid stores, or the MAC13 adenocarcinoma, which is the same histological type, but which grows without an effect on host body weight or lipid stores. While oxidation of D-[U-14C]glucose did not differ between animals bearing tumours of either type and non-tumour bearing controls, oxidation of [1-14C]triolein administered by intragastric intubation was significantly (P less than 0.05) higher in animals bearing the MAC16 tumour than in either non tumour-bearing controls or in animals bearing the MAC13 tumour. Intestinal absorption of [14C]lipid was significantly (P less than 0.05) reduced in animals bearing the MAC13 tumour when compared with either non tumour-bearing animals or MAC16 tumour-bearing animals, but was not significantly different in the latter two groups. The level of labelled lipids in heart and adipose tissue after an oral [14C]lipid load was significantly lower in animals bearing the MAC16 tumour compared with the other two groups. The level of tumour lipids was also higher in the MAC16 than in the MAC13 tumour after both an oral [14C]lipid load or by direct injection of [U-14C]palmitate complexed to albumin into epididymal fat pads. Oxidation of [U-14C]palmitate was also significantly enhanced in liver and heart homogenates from animals bearing the MAC16 tumour. These results suggest that in cachectic tumour-bearing animals mobilisation of body lipids is accompanied by an increased utilisation.


*Br. J. Cancer* (1992), **66**, 57–61 © Macmillan Press Ltd., 1992

### Lipid metabolism in cancer cachexia

H.D. Mulligan, S.A. Beck & M.J. Tisdale

*CRC Experimental Chemotherapy Group, Pharmaceutical Sciences Institute, Aston University, Birmingham B4 7ET, UK.*

**Summary** The effect of cancer cachexia on the oxidative metabolism of lipids has been studied in mice transplanted either with the MAC16 adenocarcinoma, which induces profound loss of host body weight and depletion of lipid stores, or the MAC13 adenocarcinoma, which is of the same histological type, but which grows without an effect on host body weight or lipid stores. While oxidation of D-[U-$^{14}$C]glucose did not differ between animals bearing tumours of either type and non-tumour bearing controls, oxidation of [1-$^{14}$C]triolein administered by intragastric intubation was significantly ($P<0.05$) higher in animals bearing the MAC16 tumour than in either non tumour-bearing controls or in animals bearing the MAC13 tumour. Intestinal absorption of [$^{14}$C]lipid was significantly ($P<0.05$) reduced in animals bearing the MAC13 tumour when compared with either non tumour-bearing animals or MAC16 tumour-bearing animals, but was not significantly different in the latter two groups. The level of labelled lipids in heart and adipose tissue after an oral [$^{14}$C]lipid load was significantly lower in animals bearing the MAC16 tumour compared with the other two groups. The level of tumour lipids was also higher in the MAC16 than in the MAC13 tumour after both an oral [$^{14}$C]lipid load or by direct injection of [U-$^{14}$C]palmitate complexed to albumin into epididymal fat pads. Oxidation of [U-$^{14}$C]palmitate was also significantly enhanced in liver and heart homogenates from animals bearing the MAC16 tumour. These results suggest that in cachectic tumour-bearing animals mobilisation of body lipids is accompanied by an increased utilisation.

Depletion of host fat stores is a common finding in cancer cachexia. We (Beck & Tisdale, 1987) and others (Kitada *et al.*, 1981; Masuno *et al.*, 1981) have attributed this loss of body fat, at least in part, to a circulatory lipid mobilising factor derived from the tumour cells (Beck & Tisdale, 1991). Although anorexia is common in cancer, loss of carcass fat cannot be attributed to a decreased caloric intake alone, since pair-fed rodents have been shown not to lose as much fat as tumour-bearing animals (Lundholm *et al.*, 1981). In some experimental models of cachexia, loss of host adipose tissue is associated with hyperlipidemia (Devereux *et al.*, 1984), although in the MAC16 model of cachexia, utilised in this study, plasma levels of both non esterified fatty acids (NEFA) and triacylglycerols have been shown to be reduced (Mahoney *et al.*, 1988), possibly due to an elevated level of lipoprotein lipase in skeletal muscle (Briddon *et al.*, 1991). This suggests that the liberated fatty acids are rapidly oxidised in this model. Changes in fuel utilisation have also been reported in cancer patients with lipid sources predominating (Warnold *et al.*, 1978).

In addition to host requirements the NEFA liberated during the cachectic process may also be required to maintain tumour growth. Nutritional conditions favouring mobilisation of host adipose tissue such as during an acute fast (Sauer & Dauchy, 1987*a*) and acute streptozotocin-induced diabetes (Sauer & Dauchy, 1987*b*) have been shown to stimulate tumour growth, suggesting that tumour growth *in vivo* may be limited by substances present in host fat stores. This view is strengthened by the observation that inhibition of host fat mobilisation in cancer cachexia is also associated with an inhibition of tumour growth (Tisdale & Beck, 1991). The present study documents changes of host lipid metabolism in a murine model of cachexia, the MAC16 colon adenocarcinoma. In comparison with other models this tumour induces profound weight loss in host animals with relatively small tumour burdens (>0.1% of host body weight) and without an alteration in food and water intake (Beck & Tisdale, 1987) and is useful for studying the metabolic effects of the tumour on the host in the absence of anorexia. In addition tumours of the same histological type are available which grow without an accompanying cachexia (e.g. MAC13), which can be utilised as a comparison to determine metabolic changes specific to the cachectic process.

Correspondence: M.J. Tisdale.
Received 18 December 1991; and in revised form 26 February 1991.

### Materials and methods

Pure strain NMRI mice bred in our own colony were fed a rat and mouse breeding diet (Pilsbury, Birmingham, UK) and water *ad libitum*. Fragments of either the MAC16 or MAC13 tumour were implanted into the flank of male NMRI mice (starting weight 24–26 g) by means of a trocar, as described (Bibby *et al.*, 1987). Animals bearing the MAC16 tumour developed weight loss 10–12 days following transplantation (average tumour weight 200 mg) and were used when the weight loss averaged 2 to 4 g. Animals bearing the MAC13 tumour were used 10–12 days following tumour transplantation when the average tumour weight was similar to that in animals bearing the MAC16 tumour. Non tumour-bearing mice of the same weight (26 g) were used as controls. The daily food intake in animals bearing the MAC16 tumour (15.1 ± 0.6 Kcal) did not differ from that of non tumour-bearing controls (15.3 ± 0.3 Kcal), while in animals bearing the MAC13 tumour, the daily food intake (16.4 ± 0.3 Kcal) was significantly ($P<0.01$) increased.

### *Production of $^{14}CO_2$ from D-[U-$^{14}$C]glucose*

Male NMRI mice bearing either the MAC16 tumour with weight loss (2–4 g), the MAC13 tumour or non tumour-bearing controls were injected i.v. with 50 μCi kg$^{-1}$ of D[U-$^{14}$C]glucose (sp.act. 273 mCi mmol$^{-1}$) (Amersham International, Amersham, UK) and were placed in airtight metabolic cages with the entry air being pumped through calcium carbonate (solid) to absorb any $CO_2$. Metabolically produced $^{14}CO_2$ was trapped in glass test-tubes containing 20 ml of a mixture of ethanolamine: ethoxyethanol (1:4). At specified time intervals samples (0.5 ml) were removed and the radioactivity was determined directly in Optiphase scintillation fluid (FSA Laboratory Supplies, Loughborough, UK) using a Packard Tri-Carb 2000CA liquid scintillation analyser.

### *Lipid oxidation and tissue lipid accumulation*

The absorption, accumulation and oxidation of an oral dose of [$^{14}$C]lipid was determined using the method of Oller do Nascinmento and Williamson (1986). [1-$^{14}$C]Triolein (0.33 μCi; sp.act. 114 mCi mmol$^{-1}$) (Amersham International, Amersham, Bucks, UK) together with 70 mg of non-labelled triolein was administered enterally by gastric intubation without anaesthesia to male NRMI mice bearing the MAC16 tumour and with weight loss, the MAC13 tumour or non tumour-bearing controls, with minimal stress. Immediately after administration animals were placed in airtight metabolic cages and expired $CO_2$ was collected for 5 h as described above. After 5 h, animals were anaesthetised and blood was collected by cardiac puncture. The complete gastrointestinal tract was removed and homogenised in 5 ml of 3% (w/v) $HClO_4$. Lipids were extracted from organs and blood by the method of Stansbie *et al.* (1976). The extracted fatty acids were dissolved in Optiphase scintillation fluid and the radioactivity determined as above. Triolein absorption was calculated by subtracting the total gastrointestinal tract radioactivity from that administered.

*Lipid mobilisation from direct injection of tracer into fat pads*

Serum was collected from male NRMI mice fed a 60% glucose: 40% rat and mouse breeding diet to lower their plasma NEFA level (Lyon *et al.*, 1988). [U-$^{14}$C]Palmitic acid (50 $\mu$Ci sp.act. 828 mCi mmol$^{-1}$) was dissolved in 0.5 ml of 30% (w/v) KOH and the resultant soap was dried under nitrogen. The dried soap was dissolved in 200 $\mu$l of 0.9% (w/v) NaCl and heated to 70°C until a clear solution was obtained. The soap solution was added dropwise to 200 $\mu$l of the serum, the volume of the serum being minimised so that in the final complex, the molar ratio of NEFA to serum albumin was approximately 7:1 (Lyon *et al.*, 1988). A few drops of Evans Blue dye was added to the tracer to aid visual inspection after injection to mice. Animals were anaesthetised using a mixture of halothane, oxygen and nitrous oxide (halothane 2.5%, oxygen 0.5 ml min$^{-1}$, $N_2O$ 0.7 ml min$^{-1}$). A small incision was made in the lower abdomen and the left epididymal fat pad was gently externalised using a saline wetted probe. With the aid of the magnifying glass, 2 $\mu$l of the tracer: albumin complex was injected into the fat pad using a Hamilton microsyringe. The fat pad was examined to ensure that no leakage of tracer had occurred. For time points beyond time zero the fat pad was gently returned to the abdominal cavity and the wound was clipped. At specified time points animals were sacrificed, tissues were removed and the concentration of labelled lipids determined by the methods of Stansbie *et al.* (1976).

*Oxidation of palmitate by tumour and host tissues*

Tissues were homogenised in 250 mM mannitol (500 mg tissue per ml) at 4°C and a portion of the homogenate corresponding to 100 mg of tissue was routinely incubated for up to 20 min at 37°C in a total volume of 2 ml containing 50 mM phosphate buffer, pH 7.4, 4 mM ATP, 95 mM KCl, 3 mM $MgSO_4$, 0.1 mM sodium [U-$^{14}$C]palmitate (sp.act. 1 $\mu$Ci $\mu$mole$^{-1}$), 1 mM NAD, 40 mg albumin and 1 mg cytochrome C. The assay was terminated by the addition of 0.5 ml of 10% perchloric acid and the $^{14}CO_2$ was trapped in centre wells containing 0.3 ml of 0.3 M NaOH. The water soluble products of palmitate oxidation were determined by extracting the radioactive lipid with four washes of petroleum and diethyl ether (95:2) (2 ml ml$^{-1}$ of incubation medium). Radioactivity was determined in Optiphase scintillation fluid as above.

*Statistical analysis*

Differences between groups were determined by one way analysis of variance followed by Tukey's test.

### Results

In order to minimise variations in the specific activity of blood glucose a bolus injection of D-[U-$^{14}$C]glucose was administered i.v. on a weight basis and production of $^{14}CO_2$ was measured over a short time period (10 min). Using such a protocol, production of $^{14}CO_2$ did not differ significantly between animals bearing either the MAC16 or MAC13 tumours and non tumour-bearing controls animals (Figure 1).

The ability of the animals to deal with administered lipid was investigated using [1-$^{14}$C]triolein, which was given by intragastric intubation, and the absorption over a 5 h period was monitored (Table I). While fat absorption in animals bearing the MAC16 tumour was not significantly different from that in non tumour-bearing controls, animals bearing the MAC13 tumour had a small, but significantly ($P < 0.05$) reduced fat absorption over the 5 h period. This suggests that mobilisation of the host lipid stores in cancer cachexia (Beck & Tisdale, 1987; 1991) is not due to defective lipid absorption, but that the tumour-bearing state may restrict gut function irrespective of the development of weight loss. The rate of oxidation of [1-$^{14}$C]triolein to $^{14}CO_2$ for the three groups is shown in Figure 2. At all time points examined animals bearing the MAC16 tumour had a higher excretion level of $^{14}CO_2$ than either non tumour-bearing animals or animals bearing the MAC13 tumour. In contrast, animals bearing the MAC13 tumour oxidised [1-$^{14}$C]triolein to $^{14}CO_2$ at a rate not significantly different from that in non tumour-bearing animals.

The pattern of distribution of labelled lipid between tumour and host organs also differed between the three groups (Table I). Thus heart and adipose labelled lipids together with total plasma lipids were significantly reduced in animals bearing the MAC16 tumour when compared either with animals bearing the MAC13 tumour or non tumour-bearing controls. The level of labelling of brain and adipose lipids was lower in both tumour-bearing states. There was no difference in urinary of faecal output in the three groups.

In order to understand further the path taken by lipids from host adipose tissue in the cachectic animal [U-$^{14}$C] palmitic acid complexed to albumin was directly injected into the epididymal fat pads. Lyon *et al.* (1988) have previously shown that triacylglycerol fatty acids in the heterogeneously labelled adipocytes reflected the behaviour of the respective fat pad triacylglycerol fatty acid. The rate of disappearance of triacylglycerol fatty acid radioactivity in non tumour-bearing animals and animals transplanted with the MAC16 and MAC13 tumours is shown in Figure 3. The recovery of the labelled palmitate at zero time was approximately 90%

**Figure 1** Production of $^{14}CO_2$ from D-[U-$^{14}$C]glucose by non tumour-bearing animals (□) and in animals bearing either the MAC13 (Δ) or MAC16 (■) tumours. Results are expressed as means ± s.e.m. for eight animals per group.

**Table I** Effects of tumour type on the absorption and metabolic fate of orally administered [1-$^{14}$C]triolein

| Group | *Absorption [$^{14}$C]lipid % administration dose/5 h* | *Tissue [$^{14}$C]lipid accumulation (% absorbed dose/5 h/g)* | | | | | | | |
|---|---|---|---|---|---|---|---|---|---|
| | | *Liver* | *Heart* | *Brain* | *Gastro-cnemius muscle* | *Kidney* | *Adipose* | *Plasma* | *Tumour* |
| Control | 96.1±0.8 | 1.19±0.35 | 0.51±0.14 | 0.11±0.04 | 0.12±0.02 | 0.64±0.11 | 0.84±0.13 | 0.06 ±0.02 | – |
| MAC16 | 94.0±0.9 | 0.98±0.14 | 0.26±0.05[b,d] | 0.053±0.007[a] | 0.10±0.03 | 0.63±0.11 | 0.22±0.03[b,c] | 0.037±0.008[a] | 0.16±0.08 |
| MAC13 | 86.4±2.8[a] | 1.29±0.37 | 0.48±0.04 | 0.043±0.007[a] | 0.13±0.02 | 0.56±0.04 | 0.52±0.11[a] | 0.049±0.008 | 0.09±0.01 |

All groups of animals were fed *ad libitum*. The results are mean values ± s.e.m. for six animals per group. Values that are significantly different from controls values are indicated by [a]$P<0.05$, [b]$P<0.001$. Values for MAC16 tumour-bearing animals that are significantly different by paired *t*-test from MAC13 tumour-bearing animals are indicated by [c]$P<0.05$; [d]$P<0.005$.

**Figure 2** Production of $^{14}CO_2$ from [1-$^{14}$C]triolein administered enterally by gastric intubation to non tumour-bearing animals (□) and to animals bearing either the MAC13 (Δ) or MAC16 (■) tumours. Results are expressed as mean±s.e.m. for six animals per group. Differences from controls are expressed as **a**, $P<0.05$; **b**, $<0.01$; **c**, $P<0.005$ and differences between MAC16 and MAC13 tumour-bearing animals are **d**, $P<0.005$ and **e**, $P<0.001$.

**Figure 3** Rate of loss of radioactivity from [U-$^{14}$C]palmitate labelled epididymal fat pads of non tumour-bearing animals (□) and animals bearing either the MAC16 (■) or MAC13 (Δ) tumours. Results are expressed as mean±s.e.m. for six animals per group per time point and differences from non tumour-bearing animals are shown as **a**, $P<0.05$ and between MAC16 and MAC13 tumour-bearing animals as **e**, $P<0.05$.

showing that very little leakage to other sites had occurred. While the amount of labelled lipid in the fat pads of control and MAC13 tumour-bearing animals did not change appreciably during the 60 min period after injection, there was a reduction of approximately 50% in animals bearing the MAC16 tumour. The appearance of labelled lipids in tumour and host organs 60 min after injection of the labelled palmitate is shown in Table II. Incorporation of radioactivity into liver at the 1 h time point was lower in both sets of tumour-bearing animals than in non tumour-bearing controls and incorporation into brain was higher, while incorporation into muscle was significantly greater in animals bearing the MAC16 tumour than in either control animals or those bearing the MAC13 tumour. The level of [$^{14}$C]lipid was significantly ($P<0.001$) higher in the MAC16 tumour than in the MAC13 tumour. This suggests utilisation of adipose tissue lipids by both host organs and tumour in cachectic tumour-bearing animals.

In order to investigate utilisation of lipid by individual organs and tumour, the rates of oxidation of [U-$^{14}$C]palmitate to $^{14}CO_2$ and water-soluble products was determined in homogenates. The rate of $^{14}CO_2$ production was linear for a 20 min period. The results presented in Table III show the major organ consuming palmitate was the liver. There was a 2-fold increase in conversion of [U-$^{14}$C]palmitate to $^{14}CO_2$ in liver and heart of animals bearing the MAC16 tumour and a more than 2-fold increase in muscle from animals bearing both the MAC16 and MAC13 tumours. Respiration from palmitate was low in both tumours, but was significantly higher in the MAC16 than in the MAC13 tumour. Conversion of palmitate to water-soluble products was also significantly enhanced in hearts from MAC16 tumour-bearing animals when compared with either controls or MAC13 tumour-bearing animals and was significantly enhanced in muscle of animals bearing both the MAC16 and MAC13 tumours. These results confirm that lipid oxidation was significantly higher in cachectic animals bearing the MAC16 tumour.

### Discussion

In view of its high calorific value, fat is an important fuel source when the metabolic demands of an organism are high,

**Table II** Effect of tumour type on the distribution of [U-$^{14}$C]palmitate 60 min after direct injection into epididymal adipose tissue

| | *Organ $^{14}$C lipid accumulation (% of injected dose)* | | | |
|---|---|---|---|---|
| Group | Muscle[a] | Liver | Brain | Tumour |
| Control | 1.0±0.1 | 3.9±0.6 | 0.5±0.1 | – |
| MAC16 | 1.7±0.1[c,e] | 2.1±0.4[b] | 1.7±0.1[d] | 1.7±0.1[e] |
| MAC13 | 0.8±0.1 | 1.7±0.6[b] | 1.5±0.1[c] | 0.1±0.1 |

[a]Combined values for the thigh plus gastrocnemius muscle from the left leg. Values represent $^{14}$C-labelled lipid (percentage of injected dose) per organ 60 min after direct injection of [U-$^{14}$C]palmitate into epidiymal adipose tissue. The results are mean values ± s.e.m. for six animals per group. Values that are significantly different from control values are indicated by [b]$P<0.05$; [c]$P<0.005$; [c]$P<0.001$ and values for MAC16 tumour-bearing animals that are significantly different from MAC13 tumour-bearing animals are indicated by [e]$P<0.001$.

**Table III** Utilisation of [U-$^{14}$C]palmitate by tumour and host organs

| | Tissue [$^{14}$C]palmitate conversion (nmole g h)$^d$ | | | | | | | | |
|---|---|---|---|---|---|---|---|---|---|
| | | Control | | | MAC13 | | | MAC16 | |
| *Organ* | $^{14}CO_2$ | *soluble* | *Total* | $^{14}CO_2$ | *soluble* | *Total* | $^{14}CO_2$ | *soluble* | *Total* |
| Liver | 40.2±5.4 | 448±34 | 488±34 | 41.6±7.4 | 514±10 | 560±20 | 112.6±8.4$^b$ | 466±34 | 572±40 |
| Heart | 21±1 | 79±9 | 90±7 | 20±2 | 85±6 | 106±7 | 42±4$^a$ | 122±6$^a$ | 165±9 |
| Muscle | 2.4±0.2 | 83±2 | 84±1 | 7.1±0.5$^b$ | 122±4$^a$ | 130±4 | 7.2±0.5$^b$ | 109±7$^a$ | 116±7 |
| Tumour | – | – | – | 1.2±0.07 | 44±6 | 45±6 | 2.28±0.33$^c$ | 45±6 | 47±6 |

$^a$$P<0.005$ from controls; $^b$$P<0.001$ from controls; $^c$$P<0.01$ from MAC13 tumour-bearing animals. $^d$Results are expressed as mean ± s.e.m. for between five and nine separate determinations for the various organ. The rate of conversion was calculated from the linear part of the curve.

and it appears to be the preferred calorie source in septic patients with and without cancer (Levinson *et al.*, 1988). We have previously reported that glucose utilisation by host organs, particularly the brain, is reduced in the tumour bearing state, to allow high glucose utilisation by the tumour (Mulligan & Tisdale, 1991*a*) and the present results confirm that respiration from glucose is not elevated in animals bearing the MAC tumours. Brain metabolism was maintained by an increased utilisation of 3-hydroxybutyrate, which is derived from lipid metabolism in the liver. This suggests an increased lipid requirement in the tumour-bearing state. We have simulated the situation after a high fat meal by an oral load of triolein to cachectic and non-cachectic tumour-bearing animals to investigate alterations in the ability of the host to deal with administered lipid. Previous studies in rats (Evans & Williamson, 1988*a*,*b*; Argiles *et al.*, 1989) have noted decreased lipid absorption and oxidation by two cytokines interleukin 1 and tumour necrosis factor alpha, while tumour burden (Walker 256 carcinoma) had no effect on lipid absorption and decreased oxidation. In the present study animals bearing the MAC13 tumour resembled more closely the effect of the cytokines with a slightly decreased lipid absorption compared with non tumour-bearing animals. In contrast animals bearing the MAC16 tumour and with weight loss between 2 and 4 g had a normal lipid absorption, but an increased oxidation of triolein compared with animals bearing the MAC13 tumour or non tumour-bearing controls. This increased utilisation was reflected in a lowered accumulation of [$^{14}$C]lipid in heart and adipose tissue, and an increased oxidation of [U$^{14}$C]palmitate in fortified homogenates of liver, heart and skeletal muscle. Despite massive mobilisation of body fat reserves (Beck & Tisdale, 1987) animals bearing the MAC16 tumour do not exhibit hypertriglyceridemia, and we have recently attributed this to an increase in the level of lipoprotein lipase in both heart and adiopose tissue (Briddon *et al.*, 1991). Thus the tissues of cachectic animals have an increased utilisation of fat as an energy source, suggesting that the overall energy requirements are higher in the cachectic state. This increased requirement for lipid is shown not only by catabolism of adipose tissue, but also by an increased conversion of glucose to lipids (Mulligan & Tisdale, 1991*b*) and by an increased [$^{14}$C]lipid accumulation by the MAC16 tumour.

This suggests that fat may be able to overcome some of the metabolic defects seen in cancer cachexia. Animals studies using a rat model system have shown that hyperalimentation utilising fat as the primary source of calories generated a more favourable host: tumour balance, when measured by the relative rates of growth of each (Buzby *et al.*, 1980). Intravenous infusion of a commercial triglyceride emulsion to patients with malignant disease resulted in a decrease in calorie deficit and a gain in body weight, which correlated with a gain of intracellular material (Waterhouse & Nye, 1961). Interestingly, the rate of removal of infused lipid from the blood was increased in patients with malignant disease paralleling the situation in the mouse model described in the present study.

The increased lipid oxidation in the cachectic state, together with the glucose requirement of the tumour, suggests an increased energy requirement, which could be met by an increased caloric intake. However, since the caloric intake in animals bearing the MAC16 tumour is not different from that found in non tumour-bearing controls, the increased metabolic demand would result in an energy deficiency leading to progressive weight loss.

This work has been supported by a grant from the Cancer Research Campaign. H.D.M. gratefully acknowledges receipt of a research studentship from the Royal Pharmaceutical Society of Great Britain. We thank Mr M. Wynter for the tumour transplantations and Miss A. Hudson for technical assistance.

### References

ARGILES, J.M., LOPEZ-SORIANO, F.J., EVANS, R.D. & WILLIAMSON, D.H. (1989). Interleukin-1 and lipid metabolism in the rat. *Biochem. J.*, **259**, 673.

BECK, S.A. & TISDALE, M.J. (1987). Production of lipolytic and proteolytic factors by a murine tumor-producing cachexia in the host. *Cancer Res.*, **47**, 5919.

BECK, S.A. & TISDALE, M.J. (1991). Lipid mobilising factors specifically associated with cancer cachexia. *Br. J. Cancer*, **63**, 846.

BIBBY, M.C., DOUBLE, J.A., ALI, S.A., FEARON, K.C.H., BRENNAN, R.A. & TISDALE, M.J. (1987). Characterization of a transplantable adenocarcinoma of the mouse colon producing cachexia in recipient animals. *J. Natl Cancer Inst.*, **78**, 539.

BRIDDON, S., BECK, S.A. & TISDALE, M.J. (1991). Changes in activity of lipoprotein lipase, plasma free fatty acids and triglycerides with weight loss in a cachexia model. *Cancer Lett.*, **57**, 49.

BUZBY, G.P., MULLEN, J.L., STEIN, T.P., MILLER, E.E., HOBBS, C.L. & ROSATO, E.F. (1980). Host-tumor interaction and nutrient supply. *Cancer*, **45**, 2940.

DEVEREUX, D.F., REDGRAVE, T.G., TILTON, M., HOLLANDER, D. & DECKERS, P.J. (1984). Intolerance to administered lipids in tumor-bearing animals. *Surgery*, **96**, 414.

EVANS, R.D. & WILLIAMSON, D.H. (1988*a*). Tissue-specific effects of rapid tumour growth on lipid metabolism in the rat during lactation and on litter removal. *Biochem. J.*, **252**, 65.

EVANS, R.D. & WILLIAMSON, D.H. (1988*b*). Tumour necrosis factor-alpha (cachectin) mimics some of the effects of tumour growth on the disposal of a [$^{14}$C]lipid load in virgin, lactating and litter-removed rats. *Biochem. J.*, **256**, 1055.

KITADA, S., HAYS, E.F. & MEAD, J.F. (1981). Characterization of a lipid mobilizing factor from tumors. *Prog. Lipid Res.*, **28**, 823.

LEVINSON, M.R., GROEYER, J.S., JEEVANANDAM, M. & BRENNAN, M.F. (1988). Free fatty acid turnover and lipolysis in septic mechancially ventilated cancer-bearing humans. *Metabolism*, **37**, 618.

LUNDHOLM, K., EDSTRÖM, S., EKMAN, L., KARLBERG, I. & SCHERSTEN, T. (1981). Metabolism in peripheral tissues in cancer patients. *Cancer Treat. Rep.*, **65**, (Suppl. 5), 79.

LYON, I., OOKHTENS, M., MONTISANO, D. & BAKER, N. (1988). Fat pat triacylglycerol fatty acid mobilization and oxidation in starving mice. *Biochim. Biophys. Acta.*, **958**, 188.

MAHONY, S.M., BECK, S.A. & TISDALE, M.J. (1988). Comparison of weight loss induced by recombinant tumour necrosis factor with that produced by a cachexia-inducing tumour. *Br. J. Cancer*, **57**, 385.

MASUNO, H., YAMASAKI, N. & OKUDA, H. (1981). Purification and characterization of lipolytic factor (toxohormone-L) from cell-free fluid of ascites sarcoma 180. *Cancer Res.*, **41**, 284.

MULLIGAN, H.D. & TISDALE, M.J. (1991*a*). Metabolic substrate utilization by tumour and host tissues in cancer cachexia. *Biochem. J.*, **277**, 321.

MULLIGAN, H.D. & TISDALE, M.J. (1991*b*). Lipogenesis in tumour and host tissues in mice bearing colonic adenocarcinomas. *Br. J. Cancer*, **63**, 719.

OLLER DO NASCIMENTO, C.M. & WILLIAMSON, D.H. (1986). Evidence for conservation of dietary lipid in the rat during lactation and the immediate period after removal of the litter. Increased oxidation of oral [1-$^{14}$C]triolein. *Biochem. J.*, **239**, 233.

SAUER, L.A. & DAUCHY, R.T. (1987*a*). Blood nutrient concentrations and tumour growth *in vivo* in rats: relationship during the onset of an acute fast. *Cancer Res.*, **47**, 1065.

SAUER, L.A. & DAUCHY, R.T. (1987*b*). Stimulation of tumor growth in adults rats *in vivo* during acute streptozotocin-induced diabetes. *Cancer Res.*, **47**, 1756.

STANSBIE, D., BROWNSEY, R.W., CRETTAZ, M. & DENTON, R.M. (1976). Acute effects *in vivo* of anti-insulin serum on rates of fatty acid synthesis and activities of acety-coenzyme A carboxylase and pyruvate dehydrogenase in liver and epididymal adipose tissue of fed rats. *Biochem. J.*, **160**, 413.

TISDALE, M.J. & BECK, S.A. (1991). Inhibition of tumour-induced lipolysis *in vitro* and cachexia and tumour growth *in vivo* by eicosapentaenoic acid. *Biochem. Pharmacol.*, **41**, 103.

WARNOLD, I., LUNDHOLM, K. & SCHERSTEN, T. (1978). Energy balance and body composition in cancer patients. *Cancer Res.*, **38**, 1801.

WATERHOUSE, C. & NYE, W.H.R. (1961). Metabolic effects of infused triglyceride. *Metabolism*, **10**, 403.